# A Transdermal Prion‐Bionics Supermolecule as a RAB3A Antagonist for Enhancing Facial Youthfulness

**DOI:** 10.1002/advs.202308764

**Published:** 2024-06-18

**Authors:** Wenjia Liu, Fan Ding, Wenguang Yang, Weiming You, Liqiang Zhang, Wangxiao He

**Affiliations:** ^1^ Department of Otorhinolaryngology Head and Neck Surgery The Second Affiliated Hospital of Xi'an Jiaotong University Xi'an 710004 China; ^2^ Institute for Stem Cell & Regenerative Medicine The Second Affiliated Hospital of Xi'an Jiaotong University Xi'an 710004 China; ^3^ Ministry of Education Key Laboratory of Surgical Critical Care and Life Support (Xi'an Jiaotong University) Xi'an 710004 China; ^4^ Department of Medical Oncology The First Affiliated Hospital of Xi'an Jiaotong University Xi'an 710061 P. R. China; ^5^ Department of Talent Highland The First Affiliated Hospital of Xi'an Jiaotong University Xi'an 710061 P. R. China; ^6^ National & Local Joint Engineering Research Center of Biodiagnosis and Biotherapy The Second Affiliated Hospital of Xi'an Jiaotong University Xi'an 710004 P. R. China

**Keywords:** anti‐wrinkle, peptides, prion bionics, protein–protein interactions, synaptic vesicle cycle

## Abstract

The mechanism research of skin wrinkles, conducted on volunteers underwent high‐intensity desk work and mice subjected to partial sleep deprivation, revealed a significant reduction in dermal thickness associated with the presence of wrinkles. This can be attributed to the activation of facial nerves in a state of hysteria due to an abnormally elevated interaction between SNAP25 and RAB3A proteins involved in the synaptic vesicle cycle (SVC). Facilitated by AI‐assisted structural design, a refined peptide called *RSI*_pep_ is developed to modulate this interaction and normalize SVC. Drawing inspiration from prions, which possess the ability to protect themselves against proteolysis and invade neighboring nerve cells through macropinocytosis, *RSI*_pep_ is engineered to demonstrate a GSH‐responsive reversible self‐assembly into a prion‐like supermolecule (*RSI*_prion_). *RSI*_prion_ showcases protease resistance, micropinocytosis‐dependent cellular internalization, and low adhesion with constituent molecules in the cuticle, thereby endowing it with the transdermic absorption and subsequent biofunction in redressing the frenzied SVC. As a facial mud mask, it effectively reduces periorbital and perinasal wrinkles in the human face. Collectively, *RSI*_prion_ not only presents a clinical potential as an anti‐wrinkle prion‐like supermolecule, but also exemplifies a reproducible instance of bionic strategy‐guided drug development that bestows transdermal ability upon the pharmaceutical molecule.

## Graphical abstract

Facial wrinkles resulting from nerve tension can be attributed to the activation of facial nerves in a state of hysteria, caused by an abnormally heightened interaction between SNAP25 and RAB3A proteins involved in the synaptic vesicle cycle (SVC). This study has developed a transdermal prion‐bionics supermolecule as an RAB3A antagonist to enhance facial youthfulness.

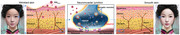

## 1 Introduction

As the fundamental building blocks of living organisms, protein–protein interactions (PPIs) are intricately involved in nearly every biological process and represent a prime target for therapeutic intervention.^[^ ^1^ ^]^ The synaptic vesicle cycle (SVC) at nerve terminals is an exemplary protein–protein interaction network that orchestrates neurotransmitter release and recovery through the processes of vesicle exocytosis, endocytosis of depleted vesicles, and regeneration of fresh ones.^[^ ^2^ ^]^ To address the persistent and imperative therapeutic needs in diseases resulting from excessive activation of peripheral nerves, including but not limited to neurodermatitis, facial wrinkles caused by nerve tension, and involuntary muscle contractions in certain areas of the face, various efforts have emerged in the exploration and development of multiple agents such as halothane anesthetics, botulinum toxins, and nucleoid genetic tools for suppressing SVC.^[^ ^3^ ^]^ Despite some achievements, achieving rapid and precise modulation of the SVC through transdermal drug delivery remains a formidable challenge, particularly for bio‐macromolecular drugs such as peptides and proteins.^[^ ^4^ ^]^

Due to the relentless pace of modern life and prolonged exposure to anxiety, an increasing number of individuals are experiencing facial furrows or creases caused by nerve tension derived from overactivated SVC.^[^ ^5^ ^]^ These facial lines, particularly noticeable around the eyes and nose, can trigger excessive distress regarding one's physical appearance and result in psychosocial challenges such as diminished work performance or a significant decline in self‐confidence.^[^ ^6^ ^]^ The improvement of the psychosocial well‐being of these individuals necessitates the urgent implementation of a straightforward yet effective therapy to address the concerns associated with these facial wrinkles. Therefore, the development of transdermal biomacromolecules to modulate overactivated SVC in facial peripheral nerves not only promotes the advancement of neurological drugs but also addresses real neuropsychological issues prevalent in today's society.

In the pursuit of this objective, an investigation was conducted on six volunteers and mice to delve into the potential mechanism underlying facial wrinkles caused by nerve tension. The study examined the subjects both before and after a demanding 28‐day period of continuous desk work for humans, as well as a challenging 28‐day period of partial sleep deprivation for mice. Astonishingly, the findings unveiled a significant reduction in dermal thickness associated with wrinkles. Subsequent investigations revealed that this dermal compression is a consequence of facial nerves being activated in a state of hysteria, which arises from an abnormally heightened interaction between SNAP25 and RAB3A proteins involved in the SVC. To alleviate these neural creases, an innovative SVC modulation was developed herein employing an AI‐designed peptide (*RSI*_pep_) with exceptional affinity for RAB3A antagonism, effectively nullifying its interaction with SNAP25. However, the remarkable biofunction of *RSI*_pep_ is impeded by the formidable barriers of cuticle adhesion, proteolysis, and cytomembrane impenetrability.

Taking inspiration from prions, which possess the ability to shield themselves against proteolysis and invade neighboring nerve cells through macropinocytosis, *RSI*_pep_ was ingeniously engineered to exhibit a GSH‐responsive reversible self‐assembly into a prion‐like supermolecule (*RSI*_prion_) that showcases remarkable resistance against proteases and relies on macropinocytosis for cellular internalization. Moreover, this prion‐like nano‐structure facilitates the *RSI*_pep_ in overcoming adhesion with constituent molecules in the cuticle, thereby endowing it with the remarkable ability to transdermally penetrate both epidermis and dermis. As a result, *RSI*_prion_ can restore the frenzied SVC to its normal state both in vitro and in vivo. Moreover, when applied as a facial mud mask with transdermal diffusion, it exhibits potent efficacy in eliminating nervous wrinkles specifically in the periorbital and perinasal region of the human face. Collectively, the report on *RSI*_prion_ not only presents a clinical translational potential for the modulation of SVC as an anti‐wrinkle prion‐like artificial protein but also exemplifies a reproducible instance of bionic strategy‐guided template‐free protein design that bestows transdermal capability upon the pharmaceutical molecule.

## 2 Results

### 2.1 The Hyperactive Interaction Between SNAP25 and RAB3A may Serve as Potential Targets for Alleviating Nervous Skin Wrinkles Derived from Hysterical SVC

**Figure 1:**
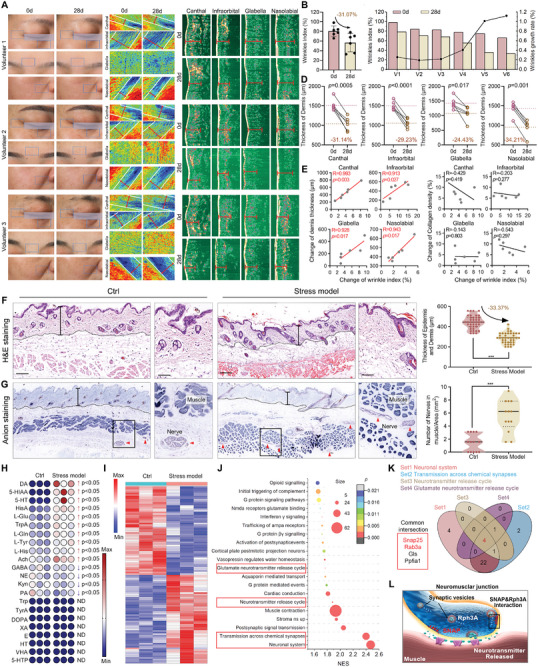
The hyperactive interaction between SNAP25 and RAB3A may serve as potential targets for alleviating nervous skin wrinkles derived from hysterical SVC. A) Wrinkles on the canthal, infraorbital, glabella, and nasolabial regions observed by VISIA before and after an arduous 28‐day period of unwavering desk work that demanded their relentless operation for over ten hours each day. B) Facial smoothness factor (wrinkles index) before and after an arduous 28‐day test. C) Thickness of the dermis in the canthal, infraorbital, glabella, and nasolabial regions. D) The relationship of wrinkles index changes to the changes of dermal thickness after 28‐day test before and in the canthal, infraorbital, glabella, and nasolabial regions. E) The H&E staining of mice skin from the stress mice model of nervous skin wrinkles, which is established through enduring a grueling 28‐day period of sleep semi‐deprivation and anxiety stimulation with electric shock. F) Nerve silver staining image of mice skin of stress model and control. G) Subcutaneous neurotransmitters release. H) Proteomics results of the skin from the stress model and control groups measured by label‐free quantitative high‐resolution mass spectrometers. I) Top 20 up‐regulated signaling pathways in GSEA analysis of the result in (H). J) Venn diagram of the four signaling pathways related to synaptic vesicle cycling in (I). The schematic diagram of SNAP25 and RAB3A function in synaptic vesicle cycling.

To investigate the impact of intensive work on facial wrinkles, the present study meticulously examined the skin wrinkle conditions of six volunteers, aged between 35 and 51 (Figure S1A, Supporting Information), who had recently returned from their winter vacation. After acquiring baseline data on canthal, infraorbital, glabella, and nasolabial wrinkles through the advanced VISIA Facial Imaging Booth (CANFIELD SCIENTIFIC, INC, USA),^[^ ^7^ ^]^ they all embarked upon an arduous 28‐day period of unwavering desk work that demanded their relentless operation for over 10 h each day. Subsequent to this period, their countenances underwent meticulous scrutiny and revealed a conspicuous augmentation in wrinkles on the canthal, infraorbital, glabella, and nasolabial regions, as evidenced by the VISIA image (**Figure** **1**A; Figure S1B, Supporting Information) and the subsequent reduction of over 30% in facial smoothness factor (wrinkles index in Figure 1B). More notably, the reduced thickness of the dermis in the canthal, infraorbital, glabella, and nasolabial regions (Figure 1C) exhibited a significant positive correlation with changes in wrinkle indexes, which is in stark contrast to the lack of statistical association observed between collagen density changes and wrinkle indexes in these areas (Figure 1D; Figure S1C, Supporting Information). This suggests that facial wrinkles in these regions are primarily caused by dermal compression rather than collagen loss.

Moreover, this dermal compression can also be observed in the stress mice model of nervous skin wrinkles, which is established through enduring a grueling 28‐day period of sleep semi‐deprivation and anxiety stimulation with electric shock (Figure S1D, Supporting Information). The dermal thickness in the skin of stress model mice, as depicted in Figure 1E, exhibited a significant reduction of over 30% compared to that of the normal‐fed control mice (Ctrl), while the collagen expression remained almost constant in both groups (Figure S1E, Supporting Information). Furthermore, the outcomes of nerve silver staining unveiled that within the control group, a majority of the nerve fibers were distributed beneath or at the periphery of the muscle layer; however, in the stress model group, a significant proportion of nerve fibers deeply infiltrated into the muscular layer (Figure 1F), thereby indicating heightened excitation of nerve cells. The reaffirmation of this finding was once again demonstrated through the detection of a cluster of neurotransmitters, wherein the secretion levels of the vast majority of detectable neurotransmitters were found to be elevated within the muscular layer of the stress model group (Figure 1G), indicative of exertion of pulling force on the muscle layer to extrude muscle layer.

To further elucidate the mechanism of epidermal nerve excitation, state‐of‐the‐art label‐free quantitative high‐resolution mass spectrometers were employed to discern proteomics in a contrasting manner between the stress model and control groups (Figure 1H). The performance of Gene Set Enrichment Analysis (GSEA) revealed the presence of four neural‐related signaling pathways among the Top 20 up‐regulated signaling pathways, as depicted in Figure 1I. The intersection algorithm was employed to analyze the constituent proteins, unveiling four common proteins, namely SNAP25, RAB3A, GLS, and PPFIA1, which exhibited up‐regulation across all four signaling pathways (Figure 1J). Among them, SNAP25 and RAB3A are directly involved in synaptic vesicle cycling (SVC) by interacting with each other to facilitate the fusion between synaptic vesicles and cytomembrane (Figure 1K).^[^ ^8^ ^]^ Therefore, we have compelling reasons to believe that antagonizing RAB3A and subsequently nullifying its interaction with SNAP25 could potentially serve as a therapeutic strategy for alleviating cutaneous wrinkles caused by hysterical superior SVC. To validate the concept, a C14‐modified membrane‐permeable siRNA targeting RAB3A was non‐invasively introduced subcutaneously into the facial region of six volunteers with wrinkles in the infraorbital and nasolabial nerve areas using electric muscle stimulation (EMS). Three days post‐treatment, all six volunteers exhibited reduced facial wrinkles, indicating the potential efficacy of RAB3A antagonism (Figure S1F, Supporting Information).

### 2.2 The RAB3A‐SNAP25 Inhibitor Peptide (*RSI*_pep_) was Discovered Through AI‐Assisted Structural Design

**Figure 2:**
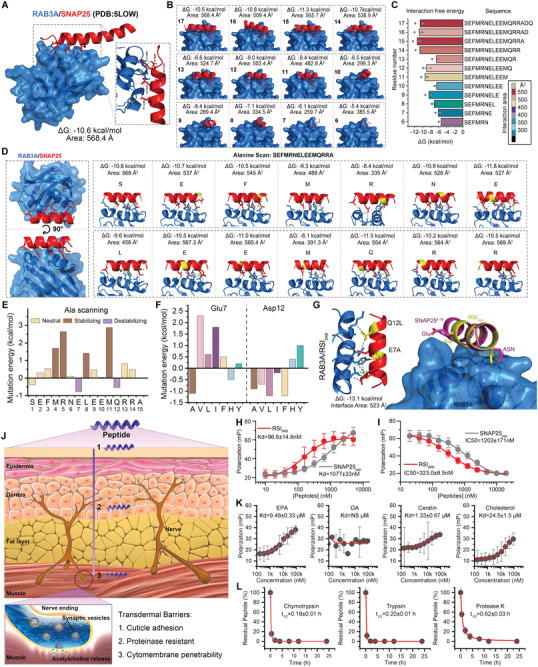
Discovery and pharmaceutical properties of RAB3A‐SNAP25 inhibitor peptide (*RSI*_pep_). A) The crystal structure of PDB code 5LOW reveals the structure foundation of the interaction between SNAP25 and RAB3A. B,C) The 17‐mer α‐helix motif derived from SNAP25 that interacts with RAB3A was systematically truncated residue by residue from its carboxyl terminal. The prediction of their binding structure with RAB3A, utilizing the remarkable Alpha Fold 2, was shown in (B) and their consequent Gibbs free energy of reaction was shown in (C). D,E) Alanine scanning of the optimal sequence from (C), characterizing by the prediction structure and consequent Gibbs free energy of reaction (E) as well as interaction area. F) The consequent Gibbs free energy of reaction in response to key binding site mutations. G) The prediction binding structure of RAB3A and *RSI*_pep_. H) The affinity test of RAB3A binding to *RSI*_pep_ or SNAP25_pep_ measured by fluorescence polarization. I) The competitive fluorescence polarization assay was conducted to authenticate the inhibitory ability of *RSI*_pep_ or SNAP25_pep_ on the interaction between SNAP25 and RAB3A. J) The schematic diagram of three formidable transdermal barriers for *RSI*_pep_. K) The affinity test of *RSI*_pep_ binding to eicosapentaenoic acid (EPA), oleic acid (OA), Ceratin, and Cholesterol measured by fluorescence polarization. L) Protease hydrolysis test of *RSI*_pep_ against the chymotrypsin, trypsin, and protease K that are present in the corium layer.

The crystal structure of PDB code 5LOW reveals that SNAP25 elegantly engages with the hydrophobic groove in RAB3A through an exquisite α‐helix motif composed of 17 amino acid residues, thereby forming a captivating complex with a binding area spanning 568.4 Å^2^ and a remarkable Gibbs free energy of reaction measuring −10.6 kcal mol^−1^ (**Figure** **2**A). The revelation of this structure has inspired us that the blockage of the SNAP25‐binding domain in RAB3A through an α‐helix peptide could potentially suppress the interaction between SNAP25 and RAB3A, thus offering a promising avenue to rewrite the SVC. To scrutinize the minimum binding sequence, a 17‐mer α‐helix motif derived from SNAP25 was systematically truncated residue by residue from its carboxyl terminal, resulting in the generation of a peptide library comprising 12 peptides (Figure 2B,C). The subsequent prediction of their binding structure with RAB3A, utilizing the remarkable Alpha Fold 2, unveils that a sequence comprising 15 amino acid residues known as SNAP25_pep_ (Seq: SEFMRNELEEMQRRA) exhibits the utmost optimal Gibbs free energy of reaction, thereby indicating an unparalleled affinity (Figure 2B,C). The identification of key binding sites in SNAP25_pep_ for RAB3A affinity was achieved through a meticulous alanine scanning approach, wherein each amino acid residue in SNAP25_pep_ was sequentially mutated to alanine (Figure 2D). The resulting mutants were subsequently subjected to binding prediction analysis employing the cutting‐edge Alpha Fold 2 algorithm (Figure 2D), wherein it was discerned that E10A and Q12A exhibited a significant reduction in the Gibbs free energy of interaction (Figure 2E). The aforementioned observation strongly suggests that E10 and Q12 serve as pivotal binding sites. Subsequently, attempts were made to mutate both E10 and Q12 into other amino acid residues, revealing that E10A and Q12L exerted the most favorable influence on RAB3A binding (Figure 2F). The subsequent prediction of the binding structure between SNAP25_pep_ E10A/Q12L (*RSI*_pep_) and RAB3A by Alpha Fold 2 validated the rationality and reliability of their optimized interaction (Figure 2G; Figure S2A, Supporting Information). To validate this, the binding affinity between FITC‐labeled *RSI*_pep_/SNAP25_pep_ and recombinant RAB3A were quantified using fluorescence polarization, revealing a remarkable over tenfold enhancement in *RSI*_pep_ binding with RAB3A compared to SNAP25_pep_ (Figure 2H). The efficacy of *RSI*_pep_, as expected, was found to be approximately fourfold greater than that of SNAP25_pep_ in inhibiting the interaction between SNAP25 and RAB3A, as determined by competitive fluorescence polarization (Figure 2I), indicating the potent potential of *RSI*_pep_ in rewriting SVC by blocking the biofunction of RAB3A.

In order to fulfill its biofunction of binding with RAB3A, *RSI*_pep_ must successfully navigate through three formidable transdermal barriers before encountering RAB3A: cuticle adhesion, proteolysis, and cytomembrane impenetrability (Figure 2J).^[^ ^9^ ^]^ Unfortunately, *RSI*_pep_ can adhere to eicosapentaenoic acid (EPA), Ceratin, and Cholesterol, all of which are essential constituents in the horny layer (Figure 2K), implying the inevitable adhesion of *RSI*_pep_ to the cuticle. To exacerbate the situation, even if a few *RSI*_pep_ manage to break through the cuticle, the chymotrypsin, trypsin, and protease K present in the corium layer would expeditiously degrade these remnants (Figure 2L). Additionally, the impenetrability of *RSI*_pep_ through the nerve cell membrane (Figure S2B, Supporting Information) was even more unfortunate, as it hindered the achievement of its biofunction in binding with RAB3A through transdermal absorption.

### 2.3 The *RSI*_pep_ is Engineered to Undergo a GSH‐Responsive Reversible Self‐Assembly, Resulting in the Formation of a Prion‐Like Supermolecule (*RSI*_prion_)

**Figure 3:**
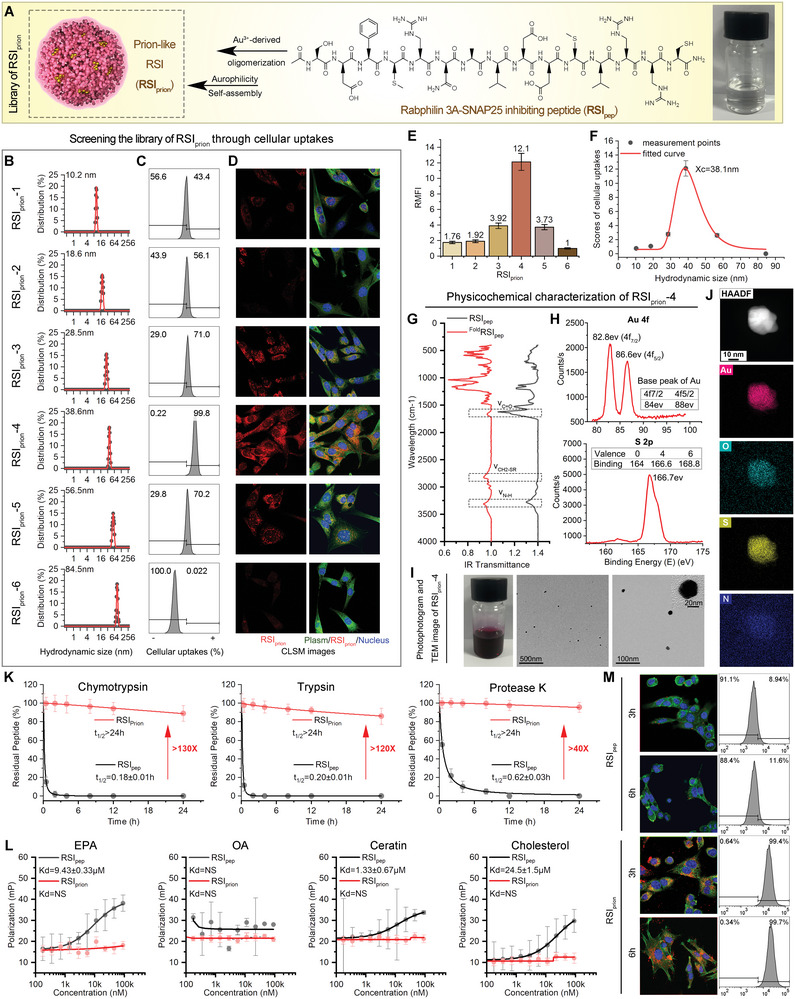
The formation of a prion‐like supermolecule (*RSI*_prion_). A) schematic diagram of *RSI*_pep_ engineering to undergo a GSH‐responsive reversible self‐assembly, resulting in the formation of a prion‐like supermolecule (*RSI*_prion_). B) hydrodynamic diameters of a diverse range of *RSI*_prion_ ranging from 10 to 85 nm measured by dynamic light scattering. C,D) Cellular uptakes of different sizes of *RSI*_prion_ into HT22 nerve cells measured by flow cytometry (FCM, C) and Laser Scanning Confocal Microscopy (LSCM, D). E) Fluorescence quantitative data of LSCM image in (D). F) The relationship between the hydrodynamic diameters of *RSI*_prion_ and the scores of cellular uptakes, as fitted by the extreme value distribution. It should be noted that the scores of cellular uptakes are determined by both FCM (C) and CLSM (E) outcomes. G) Fourier transform infrared (FT‐IR) of *RSI*_pep_ and *RSI*_prion_. H) X‐ray photoelectron spectroscopy (XPS) analysis of Au 4f and S 2p of *RSI*_prion_. I) TEM images of *RSI*_prion_. J) Elemental analysis image of S, O, N, Au overlay with one representative particle of *RSI*_prion_ taken by HRTEM. K) Protease hydrolysis test of *RSI*_pep_ and *RSI*_prion_ against the chymotrypsin, trypsin, and protease K that are present in the corium layer. L) The affinity test of *RSI*_pep_ and *RSI*_prion_ binding to eicosapentaenoic acid (EPA), oleic acid (OA), Ceratin, and Cholesterol measured by fluorescence polarization. M) Cellular uptakes of *RSI*_prion_ and *RSI*_pep_ into HT22 nerve cells measured by FCM and LSCM.

Drawing inspiration from prions, which possess the extraordinary ability to shield themselves against proteolysis and invade neighboring nerve cells through macropinocytosis, *RSI*_pep_ has been ingeniously engineered into a prion‐like supermolecule termed *RSI*_prion_ through a reversible self‐assembly with glutathione response (**Figure** **3**A).^[^ ^10^ ^]^ Through the introduction of cysteine residues (Cys) at the carboxyl terminal of *RSI*pep, the self‐assembly process is facilitated, making it possible for the Cys‐modified peptide to form oligomers with gold ions by forming a 2‐coordinate chemical link between Au(I) and free sulfhydryl in Cys.^[^ ^10^, ^11^ ^]^ The Au(I)‐peptide oligomers can subsequently undergo self‐assembly, forming exquisite supermolecules through the fascinating aurophilic interactions between Au(I) atoms.^[^ ^10^, ^11^ ^]^ By precisely adjusting the concentration of *RSI*_pep_ and pH levels within the reaction system during self‐assembly, a diverse range of *RSI*_prion_ with hydrodynamic diameters ranging from 10 to 85 nm were successfully synthesized (Figure 3B). Subsequently, succinimide activated rhodamine (Rhb‐SE) was conjugated to these *RSI*_prion_, and their uptake by HT22 nerve cells was quantified using flow cytometry (FCM) and laser scanning confocal microscopy (LSCM). The RSIprion‐4, which has a hydrodynamic diameter of 38.6 nm, representing a common size of prions,^[^ ^12^ ^]^ exhibited the highest efficiency in cellular internalization, as demonstrated by both the cellular uptake ratio measured using flow cytometry (Figure 3C) and the cellular uptake intensity measured using laser scanning confocal microscopy (Figures 3D,E). The comprehensive evaluation (product) of FCM and CLSM outcomes aligns with the extreme value distribution of the dynamic stochastic response of hydrodynamic diameter for *RSI*_prion_ (Figure 3F), indicating the superiority of *RSI*_prion_‐4, thus warranting its selection as the benchmark *RSI*_prion_ for subsequent investigations.

Based on Fourier Transform Infrared (FT‐IR) spectroscopy of *RSI*_prion_ and *RSI*_pep_, it was found that there are two absorption peaks that correspond to amide bonds at 1680 cm^−1^ (C═O) and 3300 cm^−1^ (N─H) in both infrared spectra. Furthermore, Au(I)‐SR showed a characteristic absorption peak at ≈2800 cm^−1^, indicating infinite coordination of Auric with sulfur (Figure 3G). Additionally, UV–vis spectra of *RSI*_prion_ and *RSI*_pep_ confirmed these findings by revealing characteristic absorption peaks associated with peptides and Au(I)‐SR bonds. Moreover, using X‐ray photoelectron spectroscopy (XPS) of Au4f and S2p (Figure 3H), we observed that the sulfhydryl group is coordinating with Au(I), thus confirming the existence of a coordination between Au(I) and thiolipid that forms the basis for this aurophilic self‐assembly. The successful self‐assembly of RSIprion and its uniform size with monodisperse spherical structure are subsequently revealed in transmission electron microscope (TEM) images (Figure 3I), indicating a bionic prion‐like architecture.^[^ ^13^ ^]^ Moreover, high‐resolution TEM elemental and diffraction analysis demonstrate the homogeneous distribution of gold (Au), nitrogen (N), oxygen (O), and sulfur (S) elements within *RSI*_prion_ (Figure 3J), perfectly aligning with the findings from energy dispersive X‐Ray spectroscopy (EDS) analysis results as well (Figure S3B, Supporting Information). The above‐mentioned results undeniably showcase the successful transformation of *RSI*_pep_ into a prion‐like supermolecule boasting a spherical nanostructure. The Mark‐Houwink molecular weight of this *RSI*_prion_ is ≈2938.9 KDa (Figure S3C, Supporting Information), suggesting an aggregation of ≈1400 *RSI*_pep_ molecules.

To ascertain whether *RSI*_prion_ possesses the remarkable capacity of prions to shield themselves against proteolysis, chymotrypsin, trypsin, and protease K were also employed for its treatment in a PBS buffer supplemented with essential calcium ions to activate enzymatic activity. The remarkable finding is that over 90% of *RSI*_prion_ remained intact even after a 24‐h exposure to the three proteases, while *RSI*_pep_ exhibited a significantly shorter half‐life of less than 1 h under the same conditions (Figure 3K). This can be attributed to the enhanced steric hindrance against protease recognition achieved through this engineered self‐assembly.^[^ ^14^ ^]^ Moreover, this engineered self‐assembly aids in the eradication of *RSI*_prion_’s adhesion to EPA, Ceratin, and Cholesterol, as evidenced by the nearly imperceptible change in fluorescence polarization of FITC‐labeled *RSI*_prion_ when incubated with stratum corneum material at equi‐multiplied concentration (Figure 3L). More importantly, in sharp contrast to *RSI*_pep_, *RSI*_prion_ showed significant cellular internalization into HT22 nerve cells (Figure 3M). The findings demonstrated that RSIprion not only inherited the prion's characteristic of resistance to proteolysis and ability to penetrate cytomembranes, but also possessed a low affinity for cuticles, all of which contribute to the compelling potential of *RSI*_prion_ in achieving transdermal absorption.

### 2.4 The *RSI*_prion_ is Internalized into Nerve Cells via Prion‐Like Macropinocytosis and Achieve Transdermal Absorption

**Figure 4:**
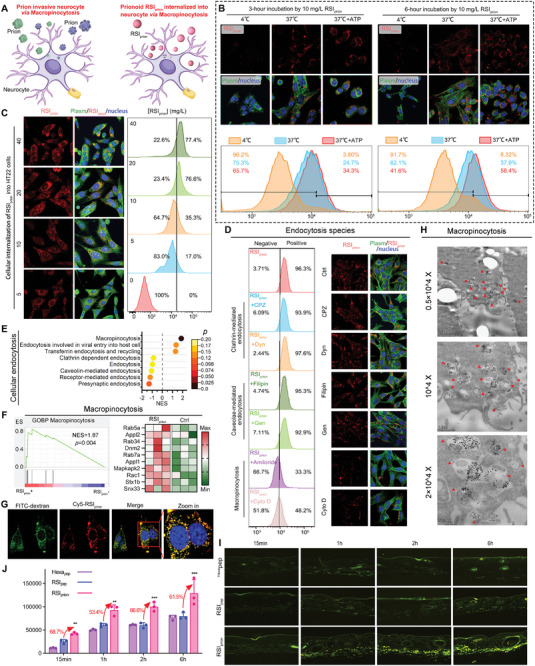
The *RSI*_prion_ is internalized into nerve cells via prion‐like micropinocytosis and achieves transdermal absorption. A) schematic diagram of prion and *RSI*_prion_ internalize into nerve cells by micropinocytosis. B) FCM and LSCM analysis of *RSI*_prion_ cellular uptakes of into HT22 cells in response to low temperature or ATP incubation. C) Cellular uptakes of *RSI*_prion_ with different concentrations into HT22 nerve cells measured by FCM and LSCM. D) FCM analysis and LSCM image of *RSI*_prion_ cellular uptakes of into HT22 cells in response to two clathrin‐mediated endocytosis inhibitor Chlorpromazine (CPZ) and Dynasore (DYN), two caveolae‐mediated endocytosis inhibitors Filipin and Genistein (GEN), as well as two macropinocytosis inhibitors Amiloride and Cytochalasin D (Cyto D). E) GSEA analysis of cellular endocytosis pathways in the proteome sequencing data of *RSI*_prion_‐treated and mock‐treated HT22 cells. F) GSEA analysis and protein levels of macropinocytosis of *RSI*_prion_‐treated and mock‐treated HT22 cells. G) The cellular uptake of *RSI*_prion_ occurred simultaneously with the internalization of 70Kd dextran, a well‐established marker for micropinocytosis, as demonstrated by the colocalization analysis conducted using LCSM. H) TEM analysis of *RSI*_prion_ cellular uptakes of into HT22 cells. The red triangle symbolizes the phagosome. I) Transdermal absorption test of FITC‐labeled ^Hexa^pep, *RSI*_pep_ and *RSI*_prion_ using the intact skin of Panama pig. J) The fluorescent quantitation results of the fluorescent image in (I).

The cunning prions skillfully exploit the intricate mechanism of macropinocytosis to infiltrate host nerve cells, while proteins or peptides aggregate into structures resembling prions, thereby cleverly stimulating macropinocytosis and facilitating their widespread dissemination into mammalian cells (**Figure** **4**A).^[^ ^15^ ^]^ To confirm the internalization of *RSI*_prion_ into nerve cells through prion‐like macropinocytosis, we initially investigated the uptake pattern of *RSI*_prion_. The cellular internalization assay during a 3‐h incubation with *RSI*_prion_ can be enhanced by ATP and inhibited by subzero treatment (Figure 4B). However, this process is not affected by concentration but is consistently efficient when the concentration exceeds 20 mg L^−1^ (Figure 4C). These findings reveal that the internalization of *RSI*_prion_ into nerve cells is an active transport mechanism rather than a passive diffusion process. To investigate the active transport species of *RSI*_prion_, a repertoire of six inhibitors representing diverse mechanisms of cell internalization were employed: two inhibitors targeting clathrin‐mediated endocytosis, Chlorpromazine (CPZ) and Dynasore (DYN); two inhibitors inhibiting caveolae‐mediated endocytosis, Filipin and Genistein (GEN); as well as two inhibitors blocking macropinocytosis, Amiloride and Cytochalasin D (Cyto D). As expected, two macropinocytosis inhibitors, Amiloride, and Cyto D, significantly suppressed *RSI*_prion_ internalization, whereas other inhibitors had no effect (Figure 4D). A GSEA analysis of cellular endocytosis pathways was performed on the proteome sequencing data of *RSI*_prion_‐treated and mock‐treated HT22 cells to provide further evidence for this outcome (Figure 4E,F). *RSI*_prion_ treatment significantly activated pathways related to macropinocytosis (Figure 4E), with all proteins in the macropinocytosis pathway exhibiting significant upregulation (Figure 4F). Additionally, the cellular uptake of *RSI*_prion_ occurred simultaneously with the internalization of 70Kd dextran, a well‐established marker for macropinocytosis,^[^ ^16^ ^]^ as demonstrated by the colocalization analysis conducted using LCSM (Figure 4G). Moreover, numerous *RSI*_prion_ supramolecular nanoparticles can be observed within a single phagophore spanning hundreds of nanometers, as evidenced by the TEM image depicting the process of *RSI*_prion_ internalization into HT22 cells (Figure 4H), which is indicative of characteristic subcellular morphological features associated with macropinocytosis.

The remarkable low cuticle adhesion, exceptional proteolysis resistance, and extraordinary cytomembrane penetrability derived from macropinocytosis all contribute to the convincing potential of *RSI*_prion_ in achieving transdermal absorption. To validate this, a comparative investigation was conducted on the intact skin of Panama Pig using three FITC‐labeled anti‐wrinkle compounds. The shin longitudinal sections in Figure 4I reveal the formidable resistance of both the commercial anti‐wrinkle peptide ^Hexa^pep and *RSI*_pep_ in penetrating the cuticle, while *RSI*_prion_ elegantly accumulates at the depths of the corium layer. The fluorescent quantitation results further elucidated this discovery, demonstrating that *RSI*_prion_ exhibited statistically significantly enhanced transdermal ability compared to ^Hexa^pep and *RSI*_pep_ (Figure 4J).

### 2.5 The *RSI*_prion_ Disassembles into *RSI*_pep_ in Response to Intracellular Glutathione and Rewrites SVC Both In Vitro and In Vivo

**Figure 5:**
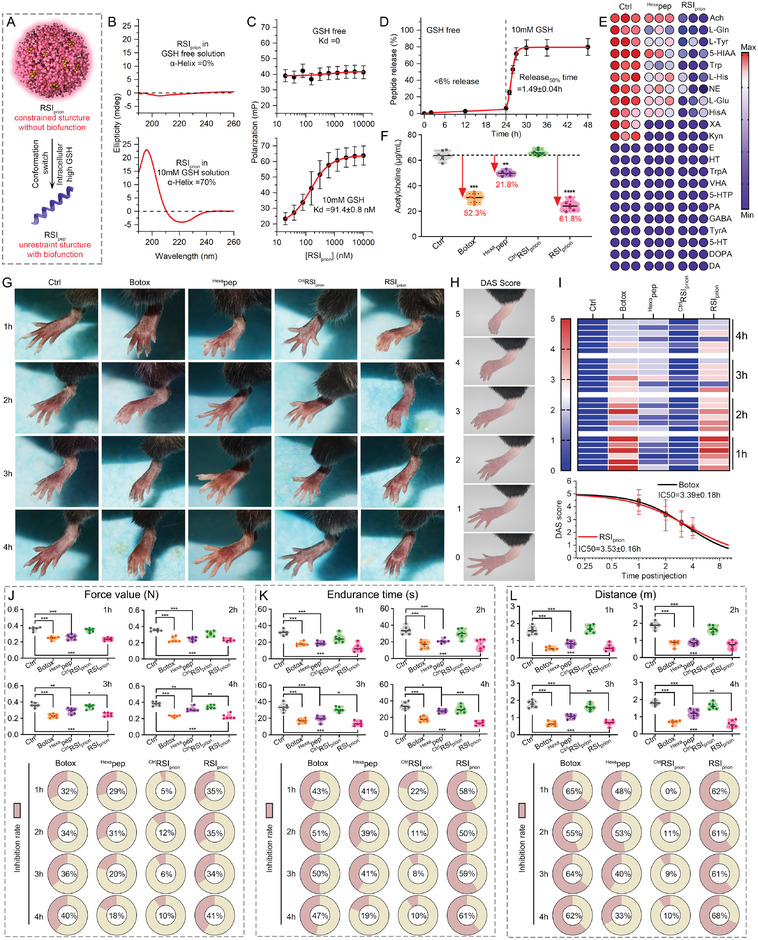
The *RSI*_prion_ disassembles into *RSI*_pep_ in response to intracellular glutathione (GSH) and rewrites SVC both in vitro and in vivo. A) The schematic diagram of *RSI*_prion_ disassembles into *RSI*_pep_ in response to intracellular glutathione. B) The circular dichroism spectrum of *RSI*_prion_ measured in buffer containing 0 mm or 10 mm GSH. C) The affinity test of *RSI*_prion_ binding to RAB3A measured by fluorescence polarization in buffer containing 0 mm or 10 mm GSH. D) The release curve of *RSI*_pep_ from *RSI*_prion_ in response to GSH. E) The neurotransmitter release from HT22 nerve cell incubating with Botox, ^Hexa^pep, *RSI*_pep_, and *RSI*_prion_. G) The experiment to authenticate the inhibition of peripheral nerves, wherein neural sedatives were administered into the musculus gastrocnemius of C57 mice (G), followed by meticulous monitoring of Digit Abduction Score (DAS) studies to ascertain the efficacy of local muscle weakening. H) Schematic of DAS scores based on paw morphology of mice. I–K) The impressive holding power (I), endurance time (J) as well as the distance covered on the rotating stick (K), in response to the injection of Botox, ^Hexa^ *pep*, *RSI*_pep_, and *RSI*_prion_.

The chemical basis of *RSI*_prion_ self‐assembly involves intramolecular coordination between Au(I)‐SR and intermolecular aurophilic interactions. Consequently, the assembly of *RSI*_prion_ can potentially be disrupted by the cleavage of Au(I)‐SR in response to a reducing environment (**Figure** **5**A). The assembled *RSI*_prion_, as depicted in Figure 5B,C, effectively shielded the intricate helical structure of *RSI*_pep_ while preserving its vital biofunction for binding with RAB3A. Remarkably, both the structural integrity and functional activity could be fully restored upon incubation with a solution containing 10 mm GSH, providing clear evidence of the disassembly process transforming *RSI*_prion_ into its monomeric form ‐*RSI*_pep_ (Figure 5B,C). The disassembly process can be further demonstrated by the release curve of *RSI*_pep_ from *RSI*_prion_, as indicated by HPLC analysis: in the presence of 10 mm GSH, free *RSI*_pep_ monomer is detected while *RSI*_prion_ remains predominantly intact even in a GSH‐free solution (Figure 5D). The disassembly of *RSI*p_rion_ in response to GSH concentration was further investigated by incubating *RSI*_prion_ with 2.5, 5, 10, and 20 µm GSH for 24 h while monitoring the release of *RSI*_pep_ using HPLC. Figure S3D (Supporting Information) demonstrates that the time required for 50% release decreased as the GSH concentration increased, indicating a concentration‐dependent mechanism for *RSI*_prion_ disassembly. The aforementioned findings have demonstrated the inherent potential of *RSI*_prion_ to undergo disassembly, thereby restoring its functional form as a monomeric entity known as *RSI*_pep_, within the intracellular reducing environment.

The biofunction of *RSI*_prion_ in rewriting SVC was investigated through a comparative study, wherein a negative control of *RSI*_prion_ called ^Ctrl^ *RSI*_prion_ was synthesized using retrosynthetic *RSI*_pep_. As anticipated, the presence of 0.02 mg mL^−1 Ctrl^ *RSI*_prion_ did not exhibit any discernible impact on the inhibition of acetylcholine release from HT22 nerve cells (Figure 5F). Conversely, the isometric *RSI*_prion_ demonstrated a remarkable ability to suppress acetylcholine release by 61.8%, surpassing both isotonic ^Hexa^pep and isopycnic commercial Botox in terms of efficacy (Figure 5F). Furthermore, *RSI*_prion_ exhibited a more comprehensive impact on the suppression of multiple neurotransmitter release in comparison to ^Hexa^pep (Figure 5E), suggesting that it restrains SVC activity rather than merely inhibiting specific neurotransmitter synthesis.

The subsequent step entailed the implementation of a classical experiment to authenticate the inhibition of peripheral nerves, wherein neural sedatives were administered into the musculus gastrocnemius of C57 mice, followed by meticulous monitoring of Digit Abduction Score (DAS) studies to ascertain the efficacy of local muscle weakening (Figure 5G,H).^[^ ^3a^ ^]^ The mice were injected with10 µg mouse^−1^ *RSI*_prion_, while ^Hexa^pep at a dosage of 10 µg mouse^−1^ served as the negative control and ^Ctrl^ *RSI*_prion_ as the positive control. Furthermore, an additional commercial positive control was used with Botox administered at a dosage of 0.05 U mouse^−1^. It is worth mentioning that the inhibitory effect on neurotransmitter release in HT22 cells by 0.05U Botox is equivalent to that of 10 µg *RSI*_prion_, which was once again confirmed by the same intramuscular median efficacy time observed for both 0.05 U mouse^−1^ Botox and 10 µg mouse^−1^ *RSI*_prion_ injection (Figure 5I). Meanwhile, ^Ctrl^ *RSI*_prion_ exhibited no efficacy in inducing local muscle weakening, while ^Hexa^pep demonstrated a limited effect with a DAS score of less than two points (Figure 5G,H). The remarkable efficacy of both Botox and *RSI*_prion_, the diminished effectiveness of ^Hexa^pep, and the complete lack of efficacy in ^Ctrl^ *RSI*_prion_ were once again demonstrated by the impressive holding power exhibited by treated mice (Figure 5J), their prolonged endurance time (Figure 5K) as well as the distance covered on the rotating stick (Figure 5L). The above results collectively demonstrate that *RSI*_prion_ effectively inhibits the excitation of peripheral nerves by modulating SVC both in vitro and in vivo.

### 2.6 The *RSI*_prion_ Boasts a Highly Commendable Safety Profile

**Figure 6:**
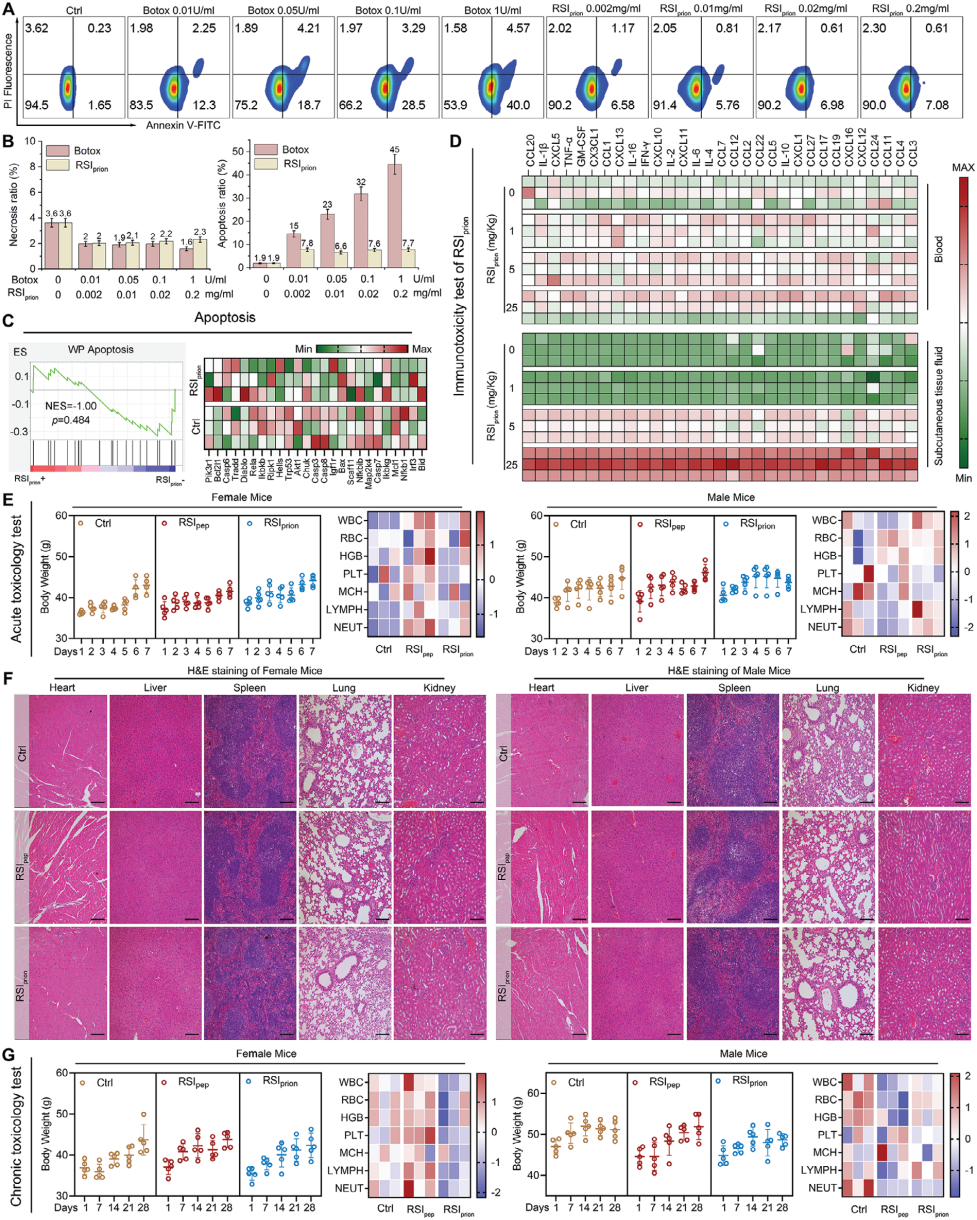
The *RSI*_prion_ boasts a highly commendable safety profile. A,B) apoptosis and necrocytosis of HT22 nerve cell induced by Botox or *RSI*_prion_ measured by FCM. C) GSEA analysis about apoptosis of the proteome from HT22 cells treated with *RSI*_prion_. D) The immunotoxicity test of *RSI*_prion_ involved subcutaneous injection of varying dosages (0, 1, 5, and 25 mg kg^−1^) into immunologically sound BALB/c mice. E,F) Acute toxicity was assessed in female and male BALB/c mice through a single subcutaneous injection of 1 mg Kg^−1^ *RSI*_prion_, *RSI*_pep_, or isopycnic normal saline. The assessment included monitoring body weights (E), conducting blood routine examinations (E), and examining representative pathological sections of organs (F). G) A 28‐day investigation of cumulative toxicity was conducted, in which subcutaneous injections of 1 mg Kg^−1^ *RSI*_prion_ or *RSI*_pep_ were administered every other day. The evaluation included monitoring body weights and conducting blood routine examinations.

The aforementioned equivalence relation states that 10 µg of *RSI*_prion_ has an equivalent effect to 0.05U of Botox. To evaluate the viability of HT22 cells, a solution containing either 10 µg of *RSI*_prion_ or 0.05U of Botox was meticulously prepared in a 50 µL cell culture medium, which was subsequently evenly diluted to administer treatment to the cells. As depicted in **Figure** **6**A,B, neither *RSI*_prion_ nor Botox induced necrocytosis of HT22 cells. Revealing a striking contrast to *RSI*_prion_, Botox demonstrated a dose‐dependent induction of apoptosis (Figure 6A,B), aligning seamlessly with the findings of the previous report.^[^ ^17^ ^]^ Fortunately, *RSI*_prion_ does not exhibit any propensity to induce apoptosis in nerve cells, as evidenced by the FCM apoptosis analysis and GSEA analysis of the proteome from HT22 cells treated with *RSI*_prion_ (Figure 6A–C).

The biosafety evaluation of *RSI*_prion_ was further enhanced by subjecting protein drugs to an immunotoxicity test, which involved subcutaneous injection of varying dosages (0, 1, 5, and 25 mg kg^−1^) into immunologically sound BALB/c mice. This was followed by immune factor detection at 2 days post‐injection. The administration of 1 mg Kg^−1^ injection, as depicted in Figure 6D, did not elicit any anaphylactic reactions in either subcutaneous tissue or blood, thereby indicating that this dosage is immunologically safe. Afterward, a meticulous assessment of acute toxicity was carried out on female and male BALB/c mice through a single subcutaneous injection of 1 mg Kg^−1^ *RSI*_prion_, *RSI*_pep_, or isopycnic normal saline. The mice treated with *RSI*_prion_ exhibited consistent body weights and blood cell indexes, showcasing the absence of any discernible impact by *RSI*_prion_ (Figure 6E). Furthermore, the biosafety of *RSI*_prion_ is once again reaffirmed through the meticulous pathological section analysis of vital organs such as heart, liver, spleen, lung, and kidney, wherein no discernible pathological alterations are observed in either *RSI*_pep_‐ or *RSI*_prion_‐treated mice (Figure 6F). The subsequent step involved a 28‐day exploration of cumulative toxicity, wherein subcutaneous injections of 1 mg Kg^−1^ *RSI*_prion_ or *RSI*_pep_ were administered every alternate day. The *RSI*_prion_ treatment, as anticipated, exhibited negligible impact on body weights and blood cell indexes (Figure 6G), thereby affirming its impeccable biosafety profile during prolonged usage.

### 2.7 The Efficacy of *RSI*_prion_ in Diminishing Facial Wrinkles Induced by Excitatory Neural Activity on the Human Countenance

**Figure 7:**
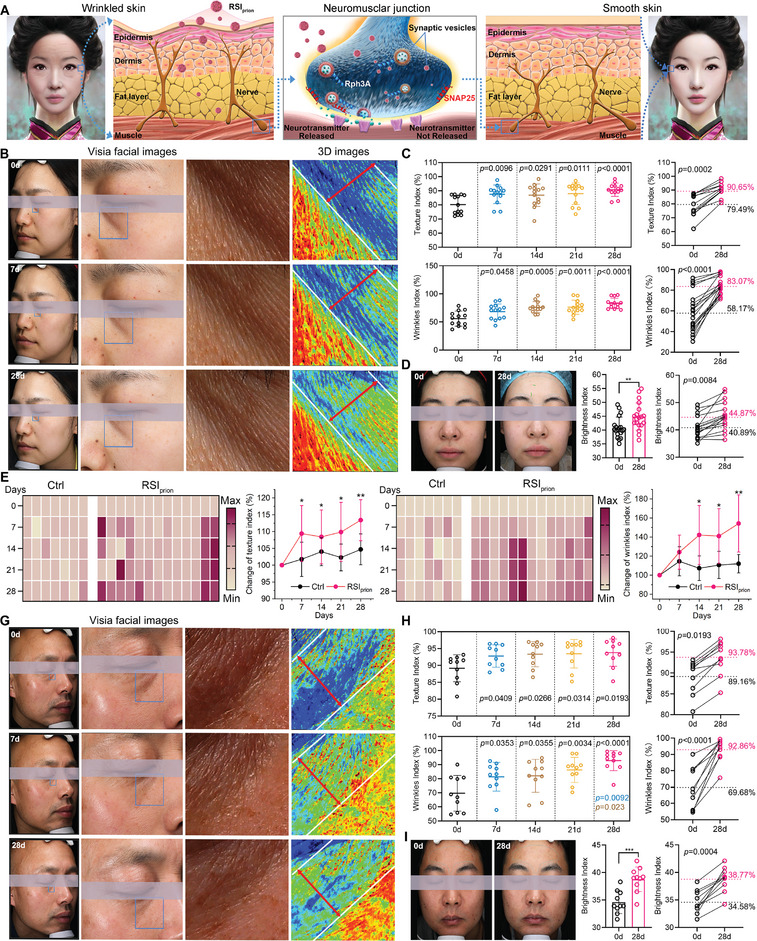
The efficacy of *RSI*_prion_ in diminishing facial wrinkles induced by excitatory neural activity on the human countenance. A) The schematic diagram of *RSI*_prion_ in diminishing facial wrinkles induced by excitatory neural activity. B) Representative facial wrinkle images observed by VISIA of a female subject treated with *RSI*_prion_ facial mud mask. C) Texure index and facial smoothness factor (wrinkles index) of all female subjects treated with *RSI*_prion_ facial mud mask. The data were evaluated using VISIA, and a higher value indicates a lower wrinkle. D) Brightness index of all female subjects treated with *RSI*_prion_ facial mud mask. E,F) Texure index(E) and wrinkles index (F) all female subjects treated with *RSI*_prion_ or *RSI*_prion_‐free facial mud mask. G) Representative facial wrinkle images observed by VISIA of a male subject treated with *RSI*_prion_ facial mud mask. H) Texure index and facial smoothness factor (wrinkles index) of all male subjects treated with *RSI*_prion_ facial mud mask. I) Brightness index of all male subjects treated with *RSI*_prion_ facial mud mask.

The remarkable performance of *RSI*_prion_ in transdermal absorption, modulation of SVC, and biosafety profiles has compelled us to investigate its efficacy in reducing facial wrinkles caused by excitatory neural activity on the human countenance (**Figure** **7**A). In pursuit of this objective, *RSI*_prion_ was ingeniously transformed into a facial mud mask by seamlessly blending it with a solution of sodium hyaluronate, and delicately applying it to the visage at a dosage of 0.01 mg kg^−1^, which equates to a mere one percent of the aforementioned safe dose of *RSI*_prion_. In a small‐scale experiment involving only three volunteers, no allergic reactions such as redness, swelling, or itching were observed on their skin within a week of use. Revealing with great excitement, the VISIA Complexion Analysis System unveiled a remarkable 22.6% increase in dermal thickness and an astonishing 26.5% reduction in wrinkles (Figure S4A, Supporting Information). The wrinkles index and texture index measured by VISIA are indicators of facial smoothness, with a value of 100% indicating no wrinkles detected by the testing machine. For more comprehensive testing, a cohort of 20‐woman volunteers aged between 32 and 60, exhibiting wrinkles induced by excitatory neural activity, were randomly assigned to partake in a 4‐week double‐blind trial. The trial consisted of two groups: one using the facial mud mask with *RSI*_prion_ (*RSI*_prion_ group) and the other without *RSI*_prion_ (Ctrl group). Specifically, each subject applied the facial mud mask once a week, and their skin condition was assessed using VISIA on days 0, 7, 12, 21, and 28. The texture index and wrinkle index in *RSI*_prion_ group exhibited a significant increase at day 7 compared to the baseline measured at day 0, indicating a remarkable reduction in facial wrinkles (Figure 7B,C). The significant reduction in facial wrinkles persisted throughout the entire 28‐day monitoring period, as demonstrated by Figure 7B,C, where the mean texture index increased from 79.49% to 90.65%, and the mean wrinkles index increased from 58.17% to 83.07%. After the 28‐day administration, the brightness index in all 13 subjects in the *RSI*_prion_ group exhibited a statistically significant increase (Figure 7D), presumably attributable to the reduction of diffuse reflection resulting from the disappearance of wrinkles. Meanwhile, the remaining seven subjects in the Ctrl group exhibited no significant alterations in either texture index (Figure 7E; Figure S4B, Supporting Information) or wrinkle index (Figure 7F; Figure S4C, Supporting Information), thereby implying that the remarkable anti‐wrinkle effect observed in the *RSI*_prion_ group can be solely attributed to the biofunction of *RSI*_prion_ rather than its excipient sodium hyaluronate. Furthermore, in order to ascertain its efficacy in the male population, a group of ten male participants were enlisted for a single‐arm trial of *RSI*_prion_, mirroring the aforementioned trial conducted with female subjects. The facial mud mask derived from *RSI*prion demonstrated remarkable efficacy in reducing facial wrinkles caused by heightened neural activity across all ten individuals (Figure 7G–I). The mean texture index increased from 89.16% to 93.78%, and the mean wrinkles index increased from 69.68% to 92.86% (Figure 7H), thereby highlighting the gender‐neutral anti‐wrinkle potential of *RSI*_prion_. Furthermore, the absence of any adverse reactions such as erythema, edema, or pruritus on the skin of male and female participants in clinical trials underscores the safety and efficacy of *RSI*_prion_ in reducing facial wrinkles caused by excitatory neural activity on the human countenance.

## 3 Discussion

The present study has meticulously observed the skin wrinkle conditions of six volunteers who have recently returned from their winter vacation, throughout an arduous 28‐day period of uninterrupted desk work that has necessitated them to operate for over 10 h each day, unveiling a conspicuous reduction in dermal thickness associated with wrinkles in all participants. Interestingly, this phenomenon can also be observed in the skin of mice who endure a grueling 28‐day period of partial sleep deprivation. Further bioinformatics analysis unveiled that the emergence of this skin wrinkle, derived from dermal compression, can be attributed to the activation of facial nerves in a state of hysteria due to an aberrantly elevated interaction between SNAP25 and RAB3A proteins involved in the synaptic vesicle cycle (Figure 1). Of note, the flat, discontinuous, hydrophobic, and large interfaces of PPIs, particularly in the case of SNAP25‐RAB3A interaction, present a significant challenge in the discovery of conventional drugs (Mw×500). However, peptides that closely mimic the 3D conformation of proteins offer valuable insights into the development of PPI modulators.^[^ ^18^ ^]^ The rewriting of this frenzied SVC herein involved the ingenious discovery, facilitated by AI‐assisted structural design, of a refined peptide called *RSI*_pep_. This remarkable peptide exhibits a nanomolar affinity for binding with RAB3A, effectively nullifying its ability to interact with SNAP25.

Despite the development of peptide PPI modulators with some degree of success, there still exist two fundamental flaws in peptides that require addressing: their inadequate proteolytic stability and ineffective cellular internalization,^[^ ^19^ ^]^ both of which have also contributed to the failure of *RSI*_pep_. The susceptibility of peptides to proteolysis is a primary flaw that significantly reduces their half‐life period, leading to insufficient potency.^[^ ^14^, ^20^ ^]^ Another limitation arises from their inherent properties, such as hydrophobicity and electronegativity, which hinder their ability to penetrate cytomembranes and undergo cellular internalization.^[^ ^21^ ^]^ To address these pharmaceutical challenges, progress has been made in the development of various peptide delivery vehicles such as macromolecule micelles, liposomes, and nanomedicine.^[^ ^14^, ^21^, ^22^ ^]^ However, most of these vehicles are primarily designed to modulate PPIs in visceral or tumor cells. Unfortunately, the complex capillary network beneath the skin poses a challenge for intravenously administered peptide PPI modulators to effectively reach subcutaneous cells via blood circulation. As a result, accessible peptide PPI modulators have long been lacking for subcutaneous cells.

Drawing inspiration from prions, which elegantly orchestrate the assembly of proteins into exquisitely organized aggregates with 3D amyloid structures solely through the intricate interplay of intermolecular forces, and possess the remarkable ability to shield themselves against proteolysis and infiltrate neighboring nerve cells via macropinocytosis, *RSI*_pep_ has undergone a magnificent transformation into an exquisite GSH‐responsive reversible prion‐like nanostructure (*RSI*_prion_) that showcases extraordinary resilience against proteases and relies on macropinocytosis for cellular internalization, all while ensuring the absence of microbiological transmissibility within communities. Moreover, this prion‐like nanostructure facilitates the *RSI*_pep_ in overcoming adhesion with constituent molecules in the cuticle, thereby endowing it with the ability to penetrate both epidermis and dermis through the transappendageal pathway, which includes hair follicles, sebaceous glands, and sweat glands.^[^ ^23^ ^]^ Thus, the remarkable efficacy of *RSI*_prion_ lies in its ability to restore the frenzied SVC to a state of equilibrium, both in vitro and in vivo.

Moreover, as an exquisite modality for manipulating peripheral nerve cell protein–protein interactions (PPIs), *RSI*_prion_ offers a plethora of unparalleled advantages, encompassing the painless administration, rapid cessation, sustained release, and circumvention of first‐pass metabolism. Additionally, when compared to the widely used wrinkle‐resistant product Botox, *RSI*_prion_ demonstrates nearly identical efficacy in SVC modulation (Figure 5) and superior biosecurity (Figure 6). Furthermore, when transformed into a luxurious facial mud mask with transdermal diffusion properties, *RSI*_prion_ exhibits unrivaled potency in eradicating neural wrinkles that afflict the periorbital and perinasal regions of the human face. Nevertheless, it is well‐known that Botox can only be administered via subcutaneous injection. Collectively, the comprehensive report on *RSI*_prion_ not only presents a clinical translational potential for the rewriting strategy of the synaptic vesicle cycle as an anti‐wrinkle prion‐like artificial protein but also exemplifies a reproducible instance of bionic strategy‐guided drug development that confers transdermal capability upon the pharmaceutical molecule.

## Conflict of Interest

The authors declare no conflict of interest.

## Supporting information

Supporting Information

## Data Availability

The data that support the findings of this study are available from the corresponding author upon reasonable request.

## References

[1] a) H. Y. Lu , Q. D. Zhou , J. He , Z. L. Jiang , C. Peng , R. S. Tong , J. Y. Shi , Signal Transduction Targeted Ther. 2020, 5, 213. 10.1038/s41392-020-00315-3 PMC7511340 32968059

[2] T. C. Südhof , Nature 1995, 375, 645. 7791897 10.1038/375645a0

[3] a) R. L. Rosales , H. Bigalke , D. Dressler , Eur. J. Neurol. 2006, 13, 2; 10.1111/j.1468-1331.2006.01438.x 16417591

[4] a) L.‐Y. Long , J. Zhang , Z. Yang , Y. Guo , X. Hu , Y. Wang , J. Drug Delivery Sci. Technol. 2020, 60, 102007;

[5] a) S. H. Lim , H. Kathuria , M. H. B. Amir , X. Zhang , H. T. T. Duong , P. C.‐L. Ho , L. Kang , J. Controlled Release 2021, 329, 907; 10.1016/j.jconrel.2020.10.021 33068646

[6] S. H. Lim , W. J. Tiew , J. Zhang , P. C.‐L. Ho , N. N. Kachouie , L. Kang , Biofabrication 2020, 12, 035003. 31952064 10.1088/1758-5090/ab6d37

[7] A. Goldsberry , C. W. Hanke , K. E. Hanke , J. Drugs Dermatol. 2014, 13, 1312. 25607694

[8] a) D. Grabs , M. Bergmann , M. Urban , A. Post , M. Gratzl , Eur. J. Neurosci. 1996, 8, 162; 8713460 10.1111/j.1460-9568.1996.tb01177.x

[9] a) M. R. Prausnitz , P. M. Elias , T. J. Franz , M. Schmuth , J.‐C. Tsai , G. K. Menon , W. M. Holleran , K. R. Feingold , Dermatology 2012, 3, 2065;

[10] a) S. Yan , J. Yan , D. Liu , X. Li , Q. Kang , W. You , J. Zhang , L. Wang , Z. Tian , W. Lu , W. Liu , W. He , Theranostics 2021, 11, 6833; 34093856 10.7150/thno.59020 PMC8171083

[11] R. P. Briñas , M. Hu , L. Qian , E. S. Lymar , J. F. Hainfeld , J. Am. Chem. Soc. 2008, 130, 975. 18154334 10.1021/ja076333e PMC2544625

[12] a) S. Jain , J. B. Udgaonkar , J. Mol. Biol. 2008, 382, 1228; 18687339 10.1016/j.jmb.2008.07.052

[13] a) P. K. Nandi , J. C. Nicole , J. Mol. Biol. 2004, 344, 827; 15533448 10.1016/j.jmb.2004.09.080

[14] a) W. He , J. Yan , F. Sui , S. Wang , X. Su , Y. Qu , Q. Yang , H. Guo , M. Ji , W. Lu , Y. Shao , P. Hou , ACS Nano 2018, 12, 11664; 30335959 10.1021/acsnano.8b07079

[15] a) J. P. Lim , P. A. Gleeson , Immunol. Cell Biol. 2011, 89, 836; 21423264 10.1038/icb.2011.20

[16] V. Jayashankar , A. L. Edinger , Nat. Commun. 2020, 11, 1121. 32111826 10.1038/s41467-020-14928-3 PMC7048872

[17] a) F. Tian , W. Cheng , J. Hu , S. Huang , S. Sun , Mol. Med. Rep. 2020, 22, 4351; 33000241 10.3892/mmr.2020.11501 PMC7533527

[18] a) H. Bruzzoni‐Giovanelli , V. Alezra , N. Wolff , C.‐Z. Dong , P. Tuffery , A. Rebollo , Drug Discovery Today 2018, 23, 272; 29097277 10.1016/j.drudis.2017.10.016

[19] a) W. Yang , W. Liu , X. Li , J. Yan , W. He , J. Adv. Res. 2023, 45, 59; 35667548 10.1016/j.jare.2022.05.009 PMC10006529

[20] W. He , J. Yan , W. Jiang , S. Li , Y. Qu , F. Niu , Y. Yan , F. Sui , S. Wang , Y. Zhou , L. Jin , Y. Li , M. Ji , P. X. Ma , M. Liu , W. Lu , P. Hou , Chem. Mater. 2018, 30, 7034. 32982042 10.1021/acs.chemmater.8b02572 PMC7518337

[21] H. Acar , J. M. Ting , S. Srivastava , J. L. LaBelle , M. V. Tirrell , Chem. Soc. Rev. 2017, 46, 6553. 28902203 10.1039/c7cs00536a

[22] F. Niu , J. Yan , B. Ma , S. Li , Y. Shao , P. He , W. Zhang , W. He , P. X. Ma , W. Lu , Biomaterials 2018, 167, 132. 29571049 10.1016/j.biomaterials.2018.03.025 PMC5889738

[23] a) P. Desai , R. R. Patlolla , M. Singh , Mol. Membr. Biol. 2010, 27, 247; 21028936 10.3109/09687688.2010.522203 PMC3061229

