# Supplementary material for: A Transdermal Prion‐Bionics Supermolecule as a RAB3A Antagonist for Enhancing Facial Youthfulness
Source: Adv Sci (Weinh). 2024 Jun 18;11(30):2308764. doi: 10.1002/advs.202308764 (PMC11321638; doi:10.1002/advs.202308764)
Supplement: Supplementary file 1 — Supporting Information [file ADVS-11-2308764-s001.docx]

**Supplementary materials**

**A transdermal prion-bionics supermolecule as a RAB3A antagonist for enhancing facial youthfulness**

**Wenjia Liu^1,*^, Fan Ding^1^, Wenguang Yang^2^, Weiming You^3^, Liqiang Zhang^1^,**

**Wangxiao He^2,*^**

^1^Institute for Stem Cell & Regenerative Medicine, The Second Affiliated Hospital of Xi’an Jiaotong University, Xi’an 710004, China

^2^ Department of Talent Highland, The First Affiliated Hospital of Xi’an Jiaotong University, Xi’an 710061, PR. China.

^3^National & Local Joint Engineering Research Center of Biodiagnosis and Biotherapy, The Second Affiliated Hospital of Xi'an Jiaotong University, Xi'an, 710004, PR. China.

^*^ Corresponding authors:

Email: wenjialiu@xjtu.edu.cn (W. Liu)

Email: hewangxiao5366@xjtu.edu.cn (W. He)

**Supplementary Figures**

**
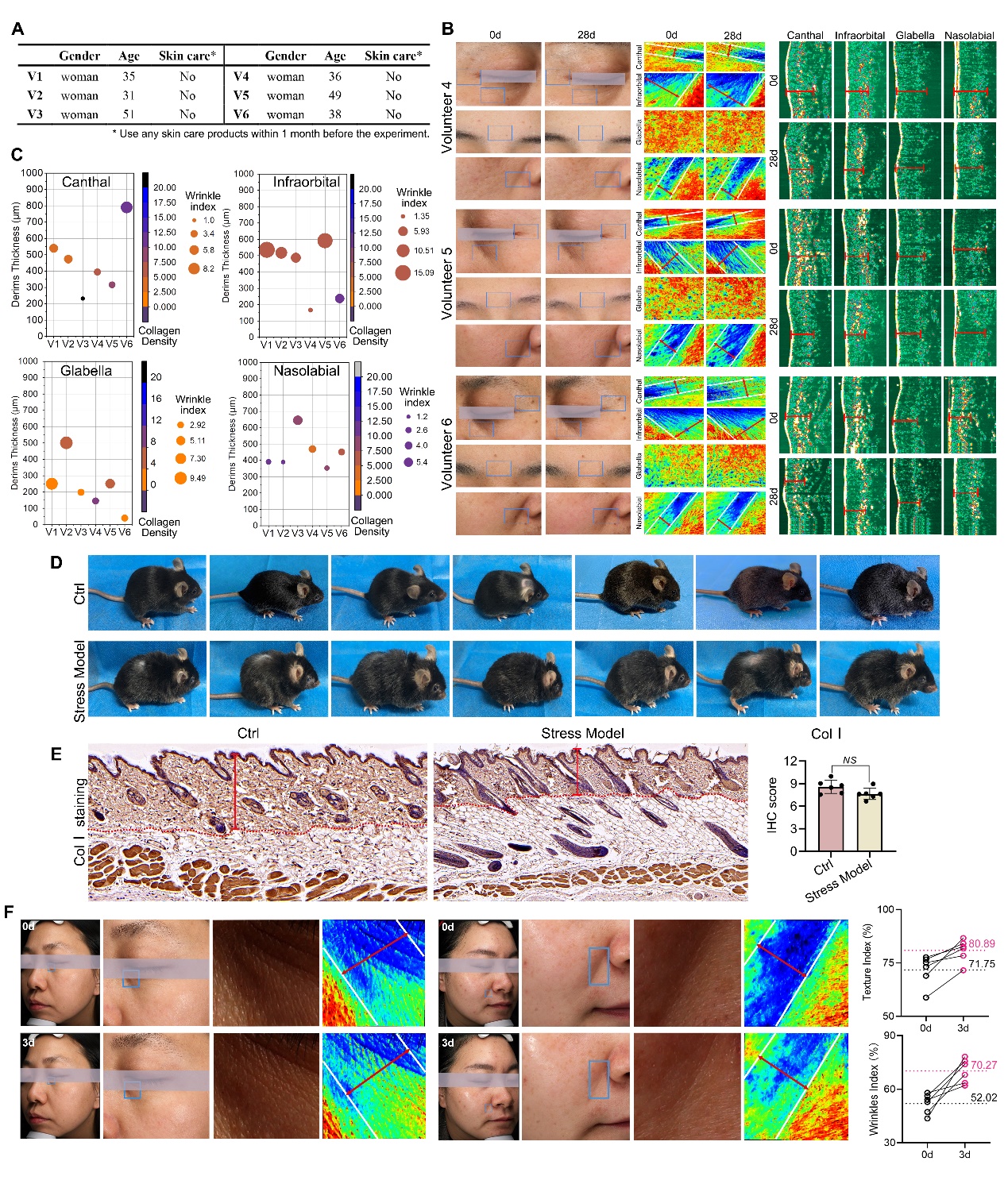
**

**Figure S1.** (**A**) General information of six volunteers who take part in research. (**B**) Wrinkles on the canthal, infraorbital, glabella, and nasolabial regions observed by VISIA before and after an arduous 28-day period of unwavering desk work that demanded their relentless operation for over ten hours each day. (**C**) Wrinkles index changes, changes of dermal thickness and collagen density change after 28-day test before and in the canthal, infraorbital, glabella, and nasolabial regions. (**D**) The photos of mice from the stress mice model of nervous skin wrinkles, which is established through enduring a grueling 28-day period of sleep semi-deprivation and anxiety stimulation with electric shock. (**E**) Collagen I staining image of mice skin of stress model and control. (F) A C14-modified membrane-permeable siRNA targeting RAB3A was non-invasively introduced subcutaneously into the facial region of six volunteers with wrinkles in the infraorbital and nasolabial nerve areas using electric muscle stimulation (EMS).


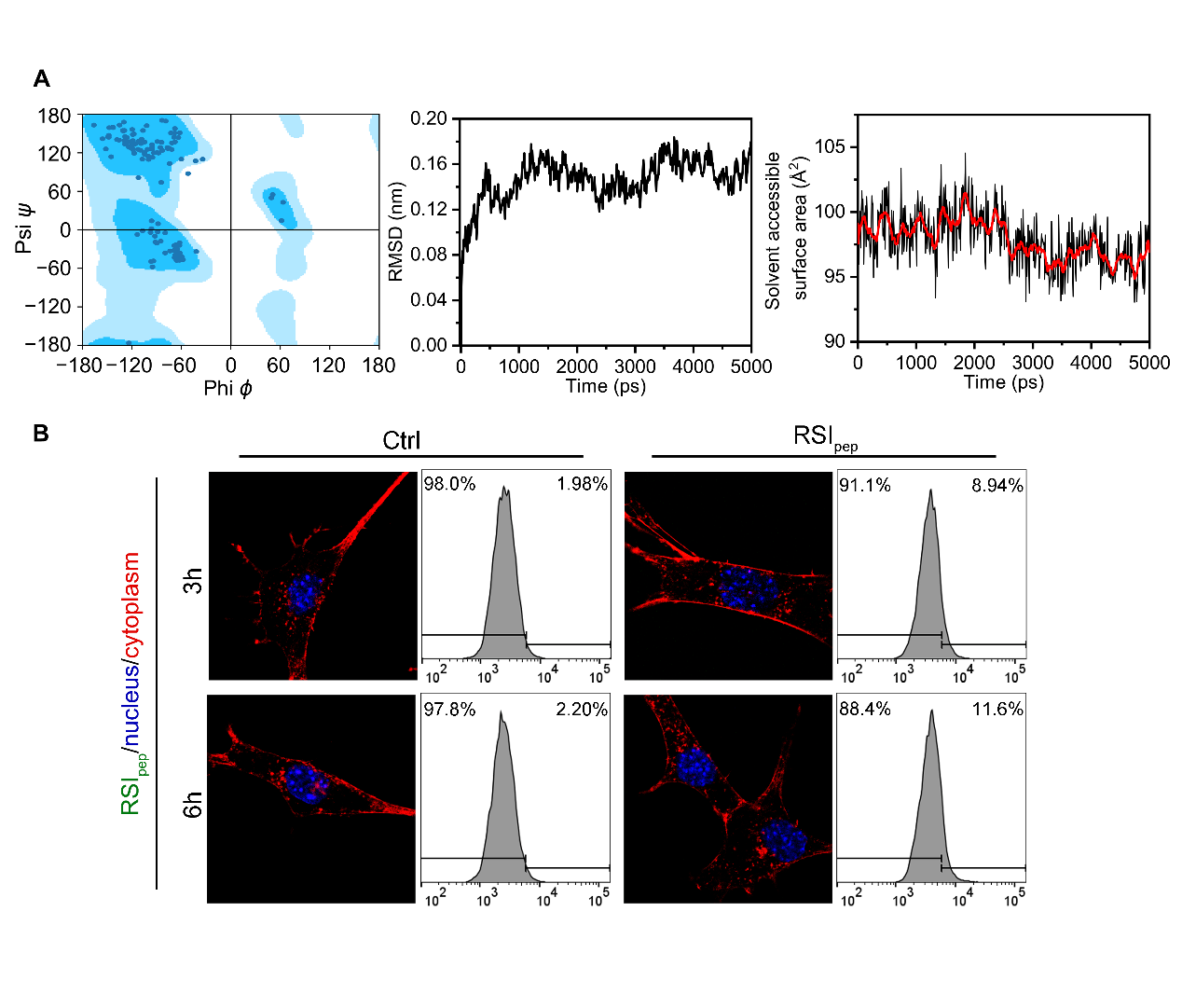


**Figure S2.** (**A**) Ramachandran plot of RSI_pep_ and RAB3A complex (the left panel), RMSD (the middle panel) and SASA (the right panel) of RSI_pep_/RAB3A complex over time in the simulation process. (**B**) Cellular uptakes of RSI_pep_ into HT22 nerve cells measured by flow cytometry and Laser Scanning Confocal Microscopy.


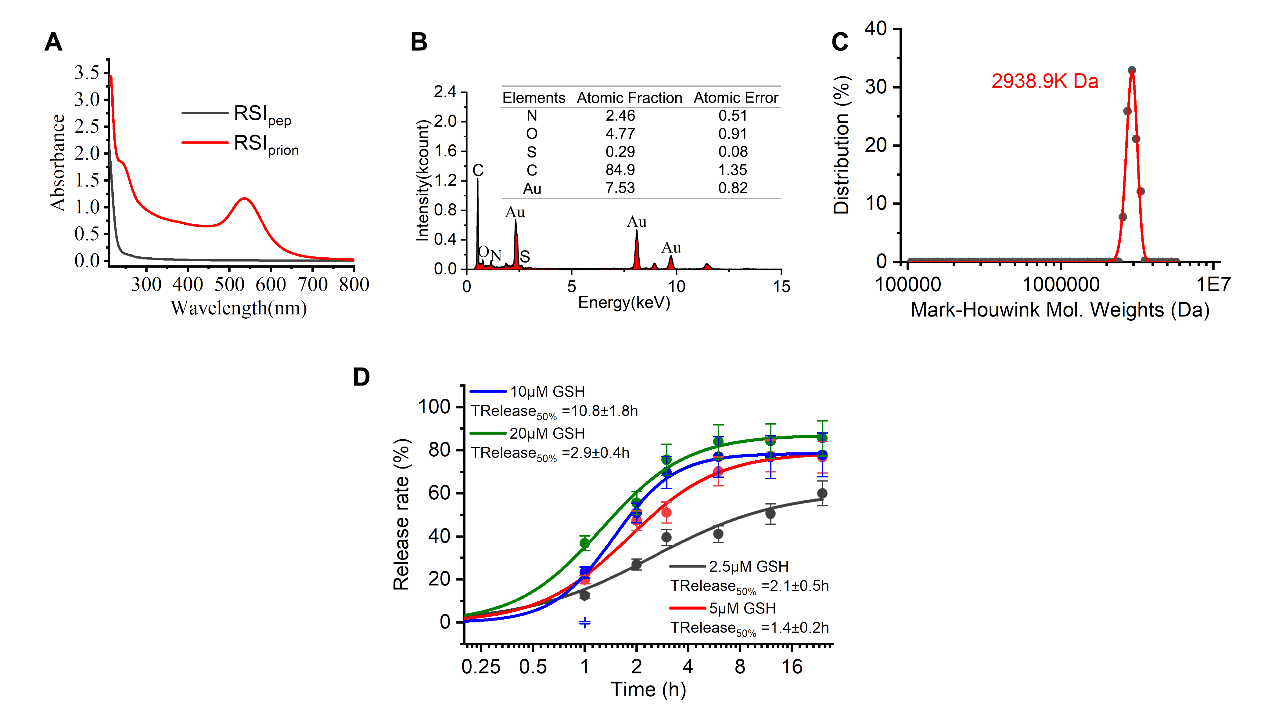


**Figure S3.** (**A**) Characterization of synthesized RSI_pep_ and RSI_prion_ by UV-Vis spectrum. (**B**) EDS quantitative element analysis of RSI_prion_. (**C**) The Mark-Houwink Mol. Weights of RSI_prion_ measured by DLS. (**D**) The release curve of RSI_pep_ from RSI_prion_ in response to 2.5μM, 5μM, 10μM, and 20μM GSH.

**
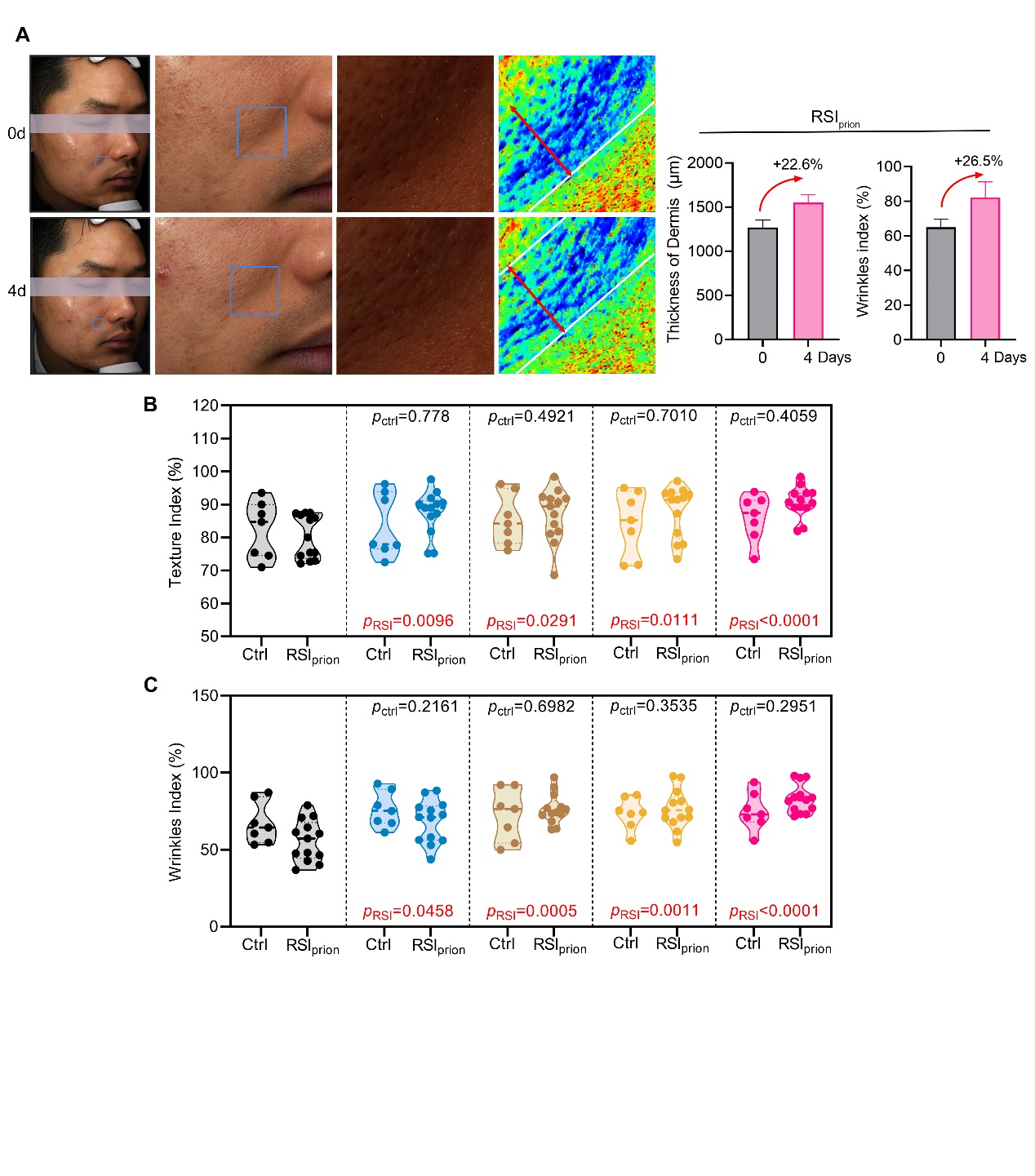
**

**Figure S4.** (**A**) Representative facial wrinkle images and dermal thickness observed by VISIA of a subject treated with RSI_prion_ facial mud mask. (**B&C**) Texure index (B) and facial smoothness factor (wrinkles index, C) of all female subjects treated with RSI_prion_-included or RSI_prion_-free facial mud mask. The data were evaluated using VISIA, and a higher value indicates a lower wrinkle.

**Experimental Section**

**General remarks**

All synthetic peptide sources were obtained from China Peptides Co.,Ltd. Phosphate buffer saline (PBS), Dulbecco’s modified Eagle’s medium (DMEM), fetal bovine serum (FBS), dimethyl sulfoxide (DMSO) and water (HPLC grade) were purchased from Fisher Scientific Ltd. HAuCl4·H2O and Tris (2-carboxyethyl) phosphine hydrochloride (TCEP) were purchased from Aladdin Chemicals. Other chemicals used in this study were purchased from Sigma-Aldrich unless otherwise specified. All products were used as received without further purification.

**Human subjects test**

The participants were health people who were recruited from Shaanxi Future Polypeptide Biotechnology Co., Ltd. Written informed consent was obtained from all participants prior to treatment. We selected the participants on the basis of several inclusion and exclusion criteria. Inclusion criteria included healthy people aged 20-55 years who were suffering from dynamic wrinkles such as nasolabial folds and forehead wrinkles. Exclusion criteria included having any allergic symptoms or autoimmune diseases; having any breakages in the skin barrier of face, neck, hands, and other test areas, or having cutaneous diseases (such as herpes); pregnant women, or women in the physiological period or breastfeeding period; taking medications or herbal preparations; having a mental illness or other conditions that make them unfit to receive treatment.

Six volunteers aged 35 to 51 were meticulously examined in order to determine the impact of intensive work on facial wrinkles. By using the VISIA Facial Imaging Booth (CANFIELD SCIENTIFIC, INC, USA), the volunteers acquired baseline data on canthal, infraorbital, glabella, and nasolabial wrinkles. In the following 28 days, they had to work tirelessly for more than ten hours a day at their desks. VISIA Facial Imaging Booth and 3D wrinkle reconstruction were used to examine facial wrinkles on day 28. In addition, skin ultrasonography was used to measure changes in dermal thickness of the skin.

In order to assess the anti-wrinkle efficacy of RSI_prion_, RSI_prion_ was applied for 7 days and 28 days in different experiment. The change in facial wrinkles of volunteers before and after RSI_prion_ application was assessed, respectively. The participants washed their faces with a mild cleanser, then dried their faces with a flake-free cleansing wipe and equilibrated in a constant humidity chamber at 22°C and 45% humidity for 20 minutes before performing a facial skin test using VISIA Facial Imaging Booth (CANFIELD SCIENTIFIC, INC, USA).

**Ethics**

All mice were purchased from the Laboratory Animal Center of Xi’an Jiaotong University. Standard chow and a typical light/dark cycle were provided to the animals under specific pathogen-free conditions. Animal experiments and volunteer tests were carried out in accordance with Institution Guidelines and approved by the Medical Experimental Ethics Committee of Xi 'an Jiaotong University (Approval number: MR-61-23-005024).

**Construction of sleep semi-deprivation model.**

Animals were stressed using the flowerpot method, first used by M. Jouvet and colleagues to modulate deprivation of REM stage of sleep, with modification [Vogel GW. A review of REM sleep deprivation. Arch Gen Psychiatry. 1975; 32:749–61. PMID:165795]. The experiment began daily at 10 a.m. A platform filled with water to 1 cm below the grid surface. The mice were individually placed on the platform and subjected to hourly timed electrical stimulation for 10 hours a day over a period of 5 consecutive days, followed by a return to normal conditions for 2 days. After 4 weeks, mice were euthanized with isoflurane to collect dorsal skin, which were washed with deionized water before fixation in 4% paraformaldehyde. The sections were cut at 5 µm thickness, deparaffinized, rehydrated, then stained with haematoxilin and eosin (H&E) according to manufacturer's protocol.

**Holmes Stain for Nerve Fibers**

Holmes staining, a silver nitrate method to detect nerve fibers and neurofibrils in tissue section, was done as described previously. Briefly, skin sections were cut at 5 µm thickness, deparaffinized and rehydrated. Then placed in 20% silver nitrate in the dark at room temperature for 2 h, and then rinsed in distilled water. The tissues were then placed in impregnating solution (1.24% Boric acid solution, 1.91% Borax solution, 1% silver nitrate, and 10% pyridine) and incubated overnight at 37°C, rinsed in distilled water and toned in 0.2% aqueous gold chloride for 4 minutes. The slides were then placed in 2% aqueous oxalic acid until the axons were thoroughly gray to black. The tissue slides were then rinsed again in distilled water, dehydrated, and mounted using a xylene base medium. Images were captured under a tissue section scanner (3DHISTECH pannoramic desk，Hungary) and analyzed *v*ia Image Pro Plus.

**Optimization of peptide sequences**

For the purpose of optimizing the length of the affinity peptide for SNP25 protein, we selected the protein complex of SNP25 and RAB3A (PDB coordinates: 5LOW), and the initial length of the affinity peptide was truncated to 17 amino acids. Subsequently, a gradual reduction in sequence length was carried out from the N-terminus of the affinity peptide, coupled with structural predictions using the AlphaFold2. Notably, the parameter of AlphaFold2 "number_relax" was set to 1, and the "template_mode" was configured as “PDB100”.Finally the PDBePISA online tool was employed to computationally assess the affinity and the interface area to determine the optimal sequence length.

**Synthesis of peptide**

All the peptides were synthesized on MBHA resin on an CS bio 336X automated peptide synthesizer with HBTU as condensation agent and DIEA as catalyst, according to the solid phase synthesis technology of Fmoc peptides. After cleavage and deprotection in a reagent cocktail containing 88% TFA, 5% phenol, 5% H_2_O and 2% TIPS, the peptides were precipitated with cold ether. Finally, after characterized by electrospray ionization mass spectrometry (ESI-MS) and HPLC, the peptides were purified to homogeneity by preparative C18 reversed-phase HPLC while the purification reagent were acetonitrile and water containing 1/1000 TFA.

**Synthesis of RSI_prion_**

2mg of RSI_pep_ peptides were fully dissolved in a solution containing 500μL PBS and 500μL ethanol through ultrasonic oscillation. After dissolution, the peptide solution was combined with 2.25 mL of HEPES (100 mM, pH 7.0), 1.25 mL of ddH20 and 500 μL of HAuCl4 (10 mM). Meanwhile, another mixture was prepared with 2.25 mL of pH 7.0 HEPES, 2.25 mL of dH2O and 500 μL of HAuCl4 (10 mM), which was then mixed with the peptide solution on a magnetic agitator at a temperature of 50℃ and a speed of 300 rpm. Finally, following a 5–10-minute reaction time and removal of excess reactants via dialysis tubing (cutoff at 10K Da), the preparation of IgP β was successfully accomplished.

**Cell culture and viability analysis**

Mouse hippocampal neuronal cell line HT22 were maintained in high glucose DMEM medium with 10% FBS. Cells were plated in 96-well plates at a density of 2000 cells/well (100 μL). After 48 h, cells were treated with ^Hexa^pep and RSI_prion_ at the indicated concentrations and times, respectively. All cells were maintained at 37°C in an atmosphere of 5% CO_2_. A standard cell viability was analyzed by Cell Counting Kit‐8 (CCK8, Beyotime, Shanghai, China) according to the manufacturer's protocols. All experiments were performed in triplicate.

**Neurotransmitter sequencing**

HT22 was cultured in 6-well plates for 24 hours and then treated either with or without ^Hexa^pep (0.02mg/mL) and RSI_prion_ (0.02mg/mL) for another 48 hours. The culture medium was collected and analyzed with an online wo-dimensional nano LC/MS/MS by BioNovoGene (Suzhou, China). In order to select the neurotranmitters that were differently expressed among the three groups, different multiples and significant levels were used (log_2_FoldChange < 0.05), after that, a Heat Map and Cluster Analysis were performed.

**RNA sequencing and analysis**

We collected dorsal skin from sleep-deprived mice as well as control mice, then isolated the RNA using the Direct-zol RNA MiniPrep Kit (Zymo Research). RNA sequencing libraries were constructed using the NEBNext® Ultra RNA Library Prep Kit for Illumina® (NEB England BioLabs). Fragmented and randomly primed 2 × 150 bp paired-end libraries were sequenced using Illumina HiSeq X Ten. Heat maps and Gene Expression Enrichment Analysis were generated using the Qlucore Omics Explorer 3.2. Pathway analysis was performed using Ingenuity Pathway Analysis (IPA) software.

**Physicochemical properties of RSI_prion_**

The CD spectra of RSI_prion_ at a concentration of 40 μM were obtained on a JASCO J-810 spectropolarimeter at room temperature in 10 mM pH 7.4 PB (pH 7.4). The morphology and lattice structure of ^RSIprion^ were observed on a high-resolution transmission electron microscope (HRTEM) (ThermoFisher Talos-F200X) operated at 200kV. One portion of the pellet was placed onto a carbon-coated copper grid for imaging with high-resolution transmission electron microscopy (HRTEM) and selected area electron diffraction (SAED).

Energy-dispersive spectroscopy (EDS) analysis was performed on the nanoparticles formed from Au^3+^ at 20 kV accelerating voltage and 133 eV resolution on a scanning area of 1 × 1 μm using an EX-250 spectrometer. The hydrodynamic size distribution (1 mg/mL in PBS, 1 mL) was obtained from the dynamic light scattering (DLS) measurement (Malvern Zetasizer Nano ZS system). For Zeta potential measurement, the nanoparticles (1 mg/mL, 1 mL) were incubated with PBS at different pH at 37 ºC for 30 min, and measured by dynamic light scattering (DLS). The surface chemical structure of modified nanocrystals was evaluated by Fourier transform infrared (FT-IR) spectroscopy (Nicolet 6700) and UV-vis absorption spectra (Shimadzu 3000 spectrophotometer).

**Fluorescence polarization (FP) assay.**

SNAP25_pep_ or RSI_pep_ was synthesized using Fmoc-chemistry SPPS and purified to homogeneity by preparative C18 RP-HPLC. FITC was conjugated to SNAP25_pep_ and RSI_pep_ *via* its N-terminal amino group in DMF, and the resultant product both FITC-SNAP25_pep_ and FITC-RSI_pep_ were HPLC-purified and lyophilized. The SNAP25_pep_ or RSI_pep_ /RAB3A binding experiments were performed in Microfluor® 2, 96-well black plates (Thermo Fisher Scientific) and readings were taken using a Tecan Infinite M2000 fluorescence plate reader. Serially diluted RAB3A proteins were prepared in Tris-HCl buffered saline (10 mM Tris, 150 mM NaCl, 1 mM EDTA, pH 7.0) and incubated with 100 nM FITC-SNAP25_pep_ and FITC-RSI_pep_ in a total volume of 150 μL per well. After a 2-hour incubation at room temperature, fluorescence polarization was measured at λex = 470 nm and λem = 530 nm. Nonlinear regression analyses were performed to give rise to K*_d_* values by using the following equation:

$$F=F_{0}+(\frac{F_{c}-F_{0}}{2\left[ Pep \right]})(\left[ Pep \right]+\left[ RAB3A \right]+K_{d}-\sqrt{{(\left[ Pep \right]+\left[ RAB3A \right]+K_{d})}^{2}-4[Pep][RAB3A]})$$

where F is measured FP, FC is FP of the SNAP25_pep_ or RSI_pep_ /RAB3A complex, F0 is FP of FITC-SNAP25_pep_ and FITC-RSI_pep_, [Pep] is the final concentration of SNAP25_pep_ or RSI_pep_, and [RAB3A] is the total concentration of RAB3A protein.

**Proteolysis resistance of RSI_prion_ and RSI_pep_**

RSI_prion_ and RSI_pep_ was dissolved in PBS solution containing 0.5 mg/ml chymotrypsin, trypsin, and protease K at a concentration of 1 mg/mL. After digestion for different time periods (0 min, 30 min, 2 h, 4 h, 8 h, 12h and 24 h), the solution was centrifuged at 14,000 g for 10 min. and then peptide in the supernatant were quantitatively detected by HPLC (instrument settings are the same as above).

**GSH-responded peptide release**

^RSIprion^ was dissolved in PBS buffer (pH 7.4) containing 5 mM glutathione (GSH), and the nanoparticles were then removed by 14000g centrifuge. Following this, the supernatants were quantified by HPLC and authenticated by ESI-MASS. Separations were performed at a flow rate of 1 mL/min with a gradient from 5 to 65% of B in 30 min (eluent A: 0.1% TFA/H_2_O, eluent B: 0.1% TFA in CH_3_CN).

**Acetylcholine content test**

HT22 was cultured in 96-well plates for 24 hours. Then, they were treated either with or without Botox (0.1U/mL), ^Hexa^pep (0.02mg/mL), RSI_pep_ (0.02mg/mL), ^Ctrl^RSI_prion_ (0.02mg/mL), RSI_prion_ (0.02mg/mL) for another 48 hours. The culture medium was collected and analyzed by using mouse Acetylcholine kits (NanJing JianCheng Bioengineering Insitute, China, A105-3-1). Acetylcholine content was determined according to the manufacturers’ instructions. All the examinations were performed in triplicate.

**Transdermal test**

Using the Franz Cell System, we evaluated the transdermal delivery of ^Hexa^pep, RSI_pep_ and RSI_prion_. To determine the efficiency of penetration, the three test samples were first labelled by FITC and then placed in contact with dorsal skin of 1-month-old pigs. The skin was collected and examined by laser confocal at different time points, including 15 minutes, 1 hour, 2 hour and 6 hours.

**Quantification of cellular uptak**e **of RSI_prion_**

HT22 cells were seeded into 96-well plates at the density of 5,000 cells per well and allowed to attach for 24 h. After that, the cells were firstly exposed to different size, ranged from 13 nm to 80nm. Then they were exposed to different concentrations of Cy5-RSI_prion_, ranged from 2.5% to 40%. After incubation at 10% concentration of nanoparticles with or without ATP (10 μM), the cells were incubation at 37℃ or 4℃ for 3 h and 6 h, washed twice with cold PBS buffer, fixed with 4% formaldehyde for 15 min. Cytoskeleton stained with Phalloidin and nuclei-stained with DAPI, then subjected to fluorescent microscopy analysis (OLYMPUS FV3000, Japan).

To study the mechanism of cellular uptake of Cy5- RSI_prion_ in HT22, the cells were pre-incubated with and different endocytosis inhibitors, including 400 μM Amiloride, 4 μg/mL filipin, 1.25 μM chlorpromazine, 1.25 μM cytochalasin D, 25 μM Genistein and 6.25 μM Dynasore for 24 h. After that, 10% concentration of Cy5- RSI_prion_ was added into each well and incubated for 6 h. The cellular uptake was qualitative analyzed by confocal microscopy and quantitative analyzed by flow cytometry (BD FACSAria Fusion, America).

**Colocalization assay under confocal microscopy**

To determine whether macropinocytosis is the key mechanism for cellular uptake of RSI_prion_ in HT22, the cells were treated with 10% concentration of Cy5- RSI_prion_ at 37 ℃ for 2 h in the presence of 1 mg/mL FITC-labelled high-molecular-mass dextran (70KDa FITC-dextran) which is an established marker of macropinocytosis. Then the cells were treated as described above before the colocalization assay. Images were captured under a confocal microscope (OLYMPUS FV3000, Japan) and analyzed via Image Pro Plus. The total particle area per cell was determined from at least three fields that were randomly selected from different regions across the entirety of each sample.

**TEM analysis of macropinocytosis**

To examine the ultrastructure of cellular uptake of RSI_prion_ in HT22, the cells were incubated with 10% concentration of RSI_prion_ for 3 h. After that, the cells were washed with PBS for two times, fixed with 2.5% glutaraldehyde at 4 C for 2 h, scratched off from the flask, centrifuged at 1500 r.p.m. for 5 min with the pellet re-suspended in 2.5% glutaraldehyde, stored at 4 ℃ until post-fixation in 1% OsO_4_ in 1 M PB, and finally subjected to ultra-thin section and microscopic analysis under a Thermo Fisher electron microscope (Scientific Talos L120C G2, America).

**Animal injections and testing**

All animal experiments were planned in accordance with Institution Guidelines, and approved by the Laboratory Animal Center of Xi'an Jiaotong University (2021-1736). Female C57/BL6 mice were anesthetized intraperitoneally with 1% pentobarbital sodium. The mice were randomly divided into six groups including control, Botox, ^Hexa^pep, RSI_pep_, ^Ctrl^RSI_prion_ and RSI_prion_ groups (n = 6 per group).

We dissolved Botox^@^ (Botulinum Toxin Type A, Allergan, Co. Mayo, Ireland), ^Hexa^pep, and RSI_pep_ in saline at a final concentration of 0.02U/100μL and 20μg/100μL, respectively. Under anesthesia with an intraperitoneal injection of pentobarbital, animals were injected with 100μL Botox, ^Hexa^pep and RSI_pep_ saline solutions, as well as 100μL ^Ctrl^RSI_prion_ and RSI_prion_ in the gastrocnemius muscle of each leg. To avoid muscle swelling caused by a single injection, 100uL of solution was injected at four points around the gastrocnemius muscle. As with the experiment groups, controls received an equivalent volume of saline injections. Mice paw morphology (DAS score), grip force test (KEW BASIS, China, KW-ZL-1), and fatigue tests (KW-6C) were performed at 1 hour, 2 hours, 3 hours and 4 hours after injection to detect muscle weakness in the mice' limbs.

**Toxicity studies**

In the 7 days following a single 500μL injection, RSI_pep_ (1mg/kg) and RSI_prion_'s (1mg/kg) acute toxicities were assessed by monitoring body weight and measuring histological and organ function indexes. To assess potential toxicities of repeatedly injecting RSI_pep_ (1mg/kg) and RSI_prion_ (1mg/kg), the animals in each group were contaminated with four intraperitoneal injections, each at an interval of 7 days, for a total of 28 days. Each mouse was experimentally administered at a dose of 200μL for once. After that, we monitored body weight of all mice over the course of treatment and measured hematological indexes as well as organ function indexes. After that, we monitored body weight of all mice over the course of treatment and measured hematological indexes as well as organ function indexes. Control mice were injected with the same dose of saline. Mice were anesthetized with 1% pentobarbital sodium, and blood was collected for complete blood count (CBC) determinations, including a white blood cell (WBC) count with differential, a red blood cell (RBC) count, mean cell hemoglobin (MCH), haemoglobin (HGB), lymphocyte percentage (LYMPH), neutrophil percentage (NEUT) and a platelet (PLT) count. Animals were then euthanized with pentobarbitone to retrieve organs, which were washed with deionized water before fixation in 4% paraformaldehyde. The tissues were processed routinely, and sections were stained with haematoxilin and eosin (H&E).

**Statistics**

Analysis for two groups were calculated using an unpaired two-tailed Student’s t-test; comparisons of more than two groups were calculated using a one-way analysis of variance (ANOVA) with Tukey post-analysis, or log-rank test where necessary. Survival analyses and curves were performed and generated according to the Kaplan-Meier method.
